# Gut microbiota influence on lung cancer risk through blood metabolite mediation: from a comprehensive Mendelian randomization analysis and genetic analysis

**DOI:** 10.3389/fnut.2024.1425802

**Published:** 2024-09-11

**Authors:** Yizhao Du, Qin Wang, Zongmei Zheng, Hailun Zhou, Yang Han, Ao Qi, Lijing Jiao, Yabin Gong

**Affiliations:** ^1^Department of Oncology, Yueyang Hospital of Integrated Traditional Chinese and Western Medicine, Shanghai University of Traditional Chinese Medicine, Shanghai, China; ^2^Institute of Translational Cancer Research for Integrated Chinese and Western Medicine, Yueyang Hospital of Integrated Traditional Chinese and Western Medicine, Shanghai University of Traditional Chinese Medicine, Shanghai, China

**Keywords:** lung cancer, gut microbiota, metabolite, Mendelian randomization, gene, mediation analysis

## Abstract

**Background:**

Gut microbiota (GM) and metabolic alterations play pivotal roles in lung cancer (LC) development and host genetic variations are known to contribute to LC susceptibility by modulating the GM. However, the causal links among GM, metabolite, host genes, and LC remain to be fully delineated.

**Method:**

Through bidirectional MR analyses, we examined the causal links between GM and LC, and utilized two-step mediation analysis to identify potential mediating blood metabolite. We employed diverse MR methods, including inverse-variance-weighted (IVW), weighted median, MR-Egger, weighted mode, and simple mode, to ensure a robust examination of the data. MR-Egger intercept test, Radial MR, MR-PRESSO, Cochran Q test and Leave-one-out (LOO) analysis were used for sensitivity analyses. Analyses were adjusted for smoking, alcohol intake frequency and air pollution. Linkage disequilibrium score regression and Steiger test were used to probe genetic causality. The study also explored the association between specific host genes and the abundance of gut microbes in LC patients.

**Results:**

The presence of *Bacteroides clarus* was associated with an increased risk of LC (odds ratio [OR] = 1.07, 95% confidence interval [CI]: 1.03–1.11, *p* = 0.012), whereas the *Eubacteriaceae* showed a protective effect (OR = 0.82, 95% CI: 0.75–0.89, *p* = 0.001). These findings remained robust after False Discovery Rate (FDR) correction. Our mediator screening identified 13 blood metabolites that significantly influence LC risk after FDR correction, underscoring cystine and propionylcarnitine in reducing LC risk, while linking specific lipids and hydroxy acids to an increased risk. Our two-step mediation analysis demonstrated that the association between the bacterial pathway of synthesis of guanosine ribonucleotides and LC was mediated by Fructosyllysine, with mediated proportions of 11.38% (*p* = 0.037). LDSC analysis confirmed the robustness of these associations. Our study unveiled significant host genes ROBO2 may influence the abundance of pathogenic gut microbes in LC patients. Metabolic pathway analysis revealed glutathione metabolism and glutamate metabolism are the pathways most enriched with significant metabolites related to LC.

**Conclusion:**

These findings underscore the importance of GM in the development of LC, with metabolites partly mediating this effect, and provide dietary and lifestyle recommendations for high-risk lung cancer populations.

## 1 Introduction

Lung cancer (LC) remains a significant burden in contemporary societies. Recent global statistics indicate an incidence rate of 11.4% and a mortality rate of 18.0% in 2020 (1). Lung cancer is primarily divided into two main types: small-cell lung cancer (SCLC) and non-small cell lung cancer (NSCLC), with NSCLC constituting 80–85% of cases (2, 3). Currently, key risk factors for developing LC include smoking, exposure to air pollution, and certain dietary habits (4), among others. Understanding and exploring modifiable risk factors is essential in reducing lung cancer incidence, promoting early diagnosis and effective treatment.

Gut microbiota (GM) emerged as the collective term for the diverse and widespread community of microorganisms inhabiting the human gastrointestinal tract. Extensive research (5, 6) indicates that alterations in the composition, function, and host interaction of the gut microbiota are directly implicated in the pathogenesis of lung cancer. In stool samples from patients with NSCLC, there was a significant increase in the levels of *Prevotella, Lactobacillus, Rikenellaceae, Streptococcus, Enterobacteriaceae, Oscillospira*, and *Bacteroides plebeius*, in contrast to lower levels found in healthy individuals (7). Further research (8) has linked early-stage lung cancer with a significant reduction in the diversity of the gut microbiota and highlighted the elevation of specific microbes, including Bacillus and *Akkermansia muciniphila*, which may contribute to the development of LC. Furthermore, the gut microbiota’s role extends beyond its association with lung cancer pathogenesis to influencing the tumor microenvironment and treatment outcomes (9). A study on the gut microbiota of NSCLC patients undergoing anti-PD-1 therapy revealed significant differences in bacterial composition between responders and non-responders (10). Considering the significant correlation between gut microbiota and pulmonary pathology, researchers have introduced the “gut-lung axis” concept (11), underscoring the bidirectional communication between gut microbiota and lung immune cell recruitment, contributing to tumorigenesis and tumor progression (12). One of the crucial mechanisms is that different metabolites stimulate or inhibit the immune system to release various cytokines (13).

The human gut microbiota is influenced by diet, environmental factors, and lifestyle, affecting tumorigenesis. For example, a high-fat diet (HFD) can promote the expansion and colonization of potentially pathogenic bacteria, like *Fusobacteria*, reduce the intake of fermentable carbohydrates and the production of butyrate (14), shift colonocyte metabolism, increase host-derived reactive oxygen and nitrogen species (RONS), and ultimately lead to DNA damage and tumorigenesis (15). Exposure of A/J mice to a mixture of cigarette smoke carcinogens NNK and BaP triggered lung carcinogenesis, increased levels of *Actinobacteria*, *Bifidobacterium*, and *Intestinimonas*, and decreased levels of *Alistipes*, *Odoribacter*, and *Acetatifactor* (16). Despite advances in techniques like 16S rRNA sequencing and the continuous updating of data, the correlation between specific gut microbiota and lung cancer risk remains elusive due to multiple influencing factors.

The emergence of metabolomics has changed our understanding of disease mechanisms (17). By enabling metabolic reprogramming, tumor cells meet their unique bioenergetic and biosynthetic needs, resulting in alterations in the levels and types of metabolites, such as glucose, amino acids, and fatty acids (18, 19). Short-chain fatty acids (SCFAs), tryptophan metabolites, polyphenolic metabolites, and conjugated linoleic acids are known to play a protective role against CRC development (20, 21). In melanoma, SCFAs and inosine are strongly associated with disease stage and treatment efficacy (22, 23). Despite these findings, the crucial impact of microbial metabolites on lung cancer development has received only scarce attention. Existing research has indicated a significant synergistic relationship between the gut microbiome and the serum metabolic profile (24). In vivo studies on lung cancer by Hagihara et al. (25) have documented significant shifts in the gut microbiome and identified an inverse relationship between the lung and gut microbiomes in activating certain metabolic pathways, notably those related to retinol, fatty acids, and linoleic acid. In comparison to healthy individuals, lung cancer patients display distinct gut microbiome and serum metabolome profiles, with higher levels of certain glycerophospholipids and hypoxanthine in the serum (26), pointing to the possibility that metabolites are utilizable as biomarkers for lung cancer. But how do they participate in the regulation of the lungs, affect the development of lung cancer? The relevant mechanism of action remains to be systematically elucidated.

Unfortunately, the causal link between metabolites and LC remains elusive, clouded by observational study limitations like lifestyle changes post-diagnosis, medication effects, and tumor-induced metabolic changes. Ethical issues, observation periods, high financial costs, and other constraints further complicate the execution of Randomized Controlled Trials (RCTs). Additionally, the variability introduced by non-standardized patient cohorts and methodologies exacerbates the challenge of pinpointing risk-increasing metabolites. Mendelian randomization (MR), which utilizes genetic variants as instrumental variables (IVs), is a widely used technique in genetic epidemiology designed to minimize the impact of potential confounding factors (27, 28). By avoiding reverse causation bias, it supports stronger causal conclusions between exposures and clinical outcomes. Recent advancements in MR research methods have reinforced its effectiveness as a prime approach for gene-level investigations, offering a clear understanding of the links between exposures and outcomes. The role of some microorganisms in cancer development exert biological effects through various metabolic pathways. Hence, the relationship of gut miceobiota and metabolite needs to be explored (29). Mediation analysis can clearly decompose the total effect into direct and indirect effects, quantifying the correlation between GM and metabolites, thereby providing a deeper understanding of how gut microbiota influences lung cancer through metabolites. Therefore, we conducted a bidirectional MR study and two-step mediation analyses using summary statistics from the largest and most up-to-date genome-wide association studies (GWAS) of the GM, blood metabolites, and LC to dissect the associations between them.

## 2 Methods

### 2.1 Study design

**Figure 1:**
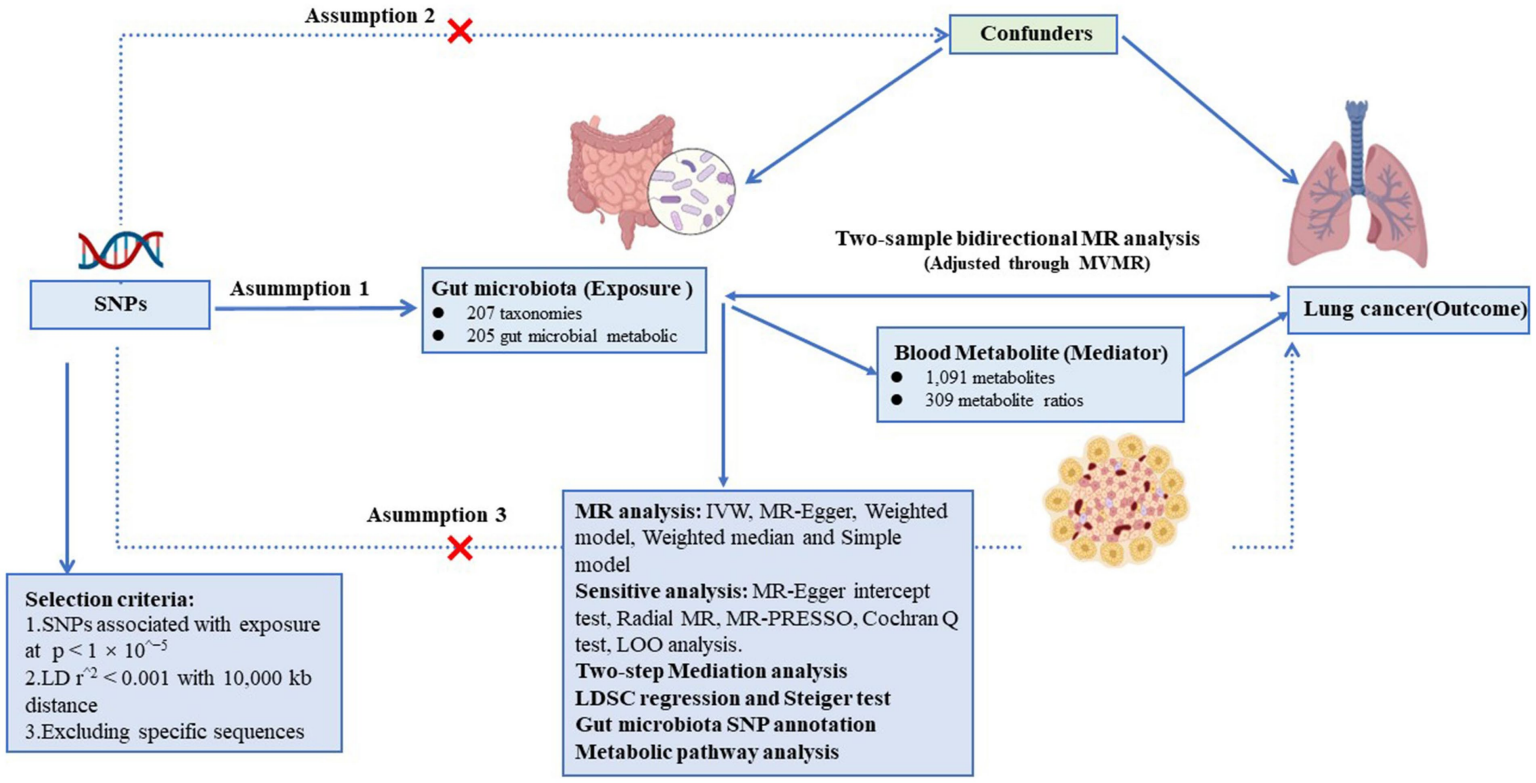
Assumptions and design of the bidirectional and mediation Mendelian randomization (MR) analyses. Firstly, a two-sample bidirectional MR was performed to investigate the causal relationships between gut microbiota and lung cancer. Then, we conducted a two-step mediation analysis to detect potential mediating metabolites (Step 1, the effect of metabolites on lung cancer; Step 2, the effect of significant gut microbiota on significant metabolites). The images for gut microbiota, blood metabolites, and lung cancer were adapted from BioRender.com.

Summary statistics on the GM, blood metabolite and LC were obtained from the respective consortia for the study design. We then explored the bidirectional two-sample MR analysis to probe the genetic causality and correlation between gut microbiota abundance and lung cancer (Figure 1). Additionally, we employed Multivariable Mendelian Randomization (MVMR) to adjust for potential confounders. And the approach we adopted for this MR analysis was grounded on 3 fundamental assumptions (Figure 1). Two-step Mediation analysis (30) was further conducted to assess the complex interactions between the GM, metabolites, and LC, pinpointing potential pathways through which gut microbiota and metabolites influence LC risk. Following this, linkage disequilibrium score regression (LDSC) was used to discern genetic associations and estimate sample overlap (31). Furthermore, instrumental variables (IVs) of GM with a direct causal association with LC were separately extracted for single nucleotide polymorphism (SNP) annotation. Finally, Significant blood metabolites related to lung cancer risk were also subject to metabolic pathway enrichment analysis. This study adhered to the reporting guidelines of the STROBE-MR (Supplementary material). Figure 1 illustrates the schematic diagram of the study design.

### 2.2 GWAS data for gut microbiota, blood metabolites and LC

The prospective cohort study LifeLines recruited 7,738 participants from the Dutch Microbiome Project (32). The summary statistics offered the most detailed insight into the genetic impact on human gut microbiota and bacterial pathways so far, identifying a comprehensive list of 207 taxonomies (comprising 5 phyla, 10 classes, 13 orders, 26 families, 48 genera, and 105 species) along with 205 gut microbial metabolic pathways. To ensure data quality, the majority of cohorts employed similar methods for interpolation and subsequent filtering. The full GWAS summary statistical data are available for download at NHGRI-EBI GWAS Catalog.^1^ Genetic data for blood metabolites also were accessed from the GWAS Catalog (see Footnote 1). Notably, this genetic data was conducted by Chen et al. (33), which was the most comprehensive analysis of genetic loci for blood metabolites thus far, identifying associations with 1,091 metabolites and 309 metabolite ratios through Genome-wide association scans with high-throughput metabolic profiling. The study encompassed a cohort of 8,299 unrelated European subjects from The Canadian Longitudinal Study of Aging (CLSA), excluding 203 European individuals identified as having first- and second-degree relatives. Among the 1,091 metabolites, 241 were defined as unknown due to as yet poorly defined chemical properties. Another 850 metabolites were chemically authenticated and allocated to eight super pathways, including lipids, amino acids, xenobiotics, nucleotides, cofactors and vitamins, carbohydrates, peptides, and energy. The analysis not only expanded the spectrum of known metabolites but also provided an in-depth exploration of metabolite ratios, which have been scarcely studied.

To bolster statistical power, validate research findings, and foster the exploration of potential new associations, summary-level GWAS data for the association analysis between the gut microbiota, metabolite and LC were obtained from two large GWAS meta-analyses. One GWAS encompassed 29,863 cases and 55,586 controls of European descent from the Transdisciplinary Research in Cancer of Lung team (TRICL) based on OncoArray and other Illumina genome-wide arrays. TRICL is a member of an organization focused on Genetic Associations and Mechanisms in Oncology (GAME-ON consortium). The data was downloaded from GWAS Catalog (see Footnote 1) (34). The other was a primary meta-analysis of the Lung Cancer Cohort Consortium (LC3), International Agency for Research on Cancer (IARC), MD Anderson Cancer Center (MDACC), St. Luke’s Radiation Research Institute (SLRI), The Institute of Cancer Research (ICR), Harvard, National Cancer Institute (NCI), Germany and deCODE, which encompassed 85,716 individuals of European descent (29,266 patients and 56,450 controls) (35). Characteristics of corresponding GWAS data sources are described in Table 1.

**Table 1:** The data source of exposure, mediator and outcome.

	Traits	Sample size	Consortium	Ancestry	Year	Download URL	Accession number
Exposure	Human Gut microbiota	7,738	doi: 10.1038/s41588-021-00992-y	European	2022	https://www.ebi.ac.uk/gwas/	GCST90027446 to GCST90027857
Mediator	Blood metabolites	8,299	doi: 10.1038/s41588-022-01270-1	European	2023	https://www.ebi.ac.uk/gwas/	GCST90200453 to GCST90200711
Outcome	Lung cancer	85,449	TRICL	European	NA	https://www.ebi.ac.uk/gwas/	ieu-a-987
	Lung cancer	85,716	doi: 10.1038/ng.3892	European	2017	https://www.ebi.ac.uk/gwas/	ebi-a-GCST004748

Notations: TRICL, Transdisciplinary Research in Cancer of Lung team.

This study utilized data that is accessible to the public. Each study involved in the GWAS was approved by the appropriate Institutional Review Board, and consent was obtained from the participants or their legal representatives, guardians, or proxies.

### 2.3 Instrumental variable selection

The criteria for selecting IVs were determined through a methodical process: (1) Threshold Adjustment: Guided by research (36, 37), we adjusted the threshold to *p* < 1 × 10^−5^ for locus-wide significance; (2) Performing a linkage disequilibrium (LD) analysis with an r^2^ < 0.001 and a clumping window of 10,000 kb; (3) Excluding palindromic sequences and SNPs with allelic discrepancies between samples; (4) Removing SNPs with an *F* value <10 to reduce bias, using R^2^ and F statistics to assess instrument quality; (5) Identified LC’s main risk factors—smoking, alcohol use, and air pollution—through literature review (4, 38), we then used the PhenoScanner database (39) to remove confounders. This culminated in an MR analysis of gut microbiota and metabolites associated with at least two SNPs. Given the evidence from previous studies highlighting the adverse effects of unhealthy lifestyles, such as smoking and drinking, along with air pollution on the respiratory system, we conducted an MVMR analysis (40) to adjust for genetic liability to the aforementioned risk factors, using the same IV screening procedures and criteria as mentioned above.

### 2.4 Statistical and sensitivity analysis

To explore the link between GM, metabolites, and LC, we employed five methods: inverse variance weighted (IVW), MR-Egger, Weighted Median (WM), Simple Model, and Weighted Model. IVW combines Wald estimates across loci for multi-SNP analysis, assuming no horizontal pleiotropy (41). MR-Egger addresses pleiotropy under the InSIDE assumption, correcting biases via weighted regression (42). The WM offers reliable causal effect estimates, especially when valid IVs contribute over 50% of the weight (43), by prioritizing larger effect sizes for consistency even with less valid SNPs. The Simple Model posits a direct causal link between genetic variants and outcomes (44), minimizing biases from complex MR methods. IVW is the primary method, with the rest providing supportive results. Consistency across methods suggests reliability. We applied an FDR correction for multiple comparisons to reduce false positives, indicating that non-significant post-correction results may still suggest a potential causal link to lung cancer.

Sensitivity analysis is vital for addressing horizontal pleiotropy and heterogeneity, which can affect MR estimates significantly. Horizontal pleiotropy occurs when IVs influence the outcome through unrelated pathways. We employed a quartet of methods to identify and adjust for heterogeneity and pleiotropy: MR-Egger intercept, Radial MR, MR-PRESSO, and the Cochran Q test (45). MR-Egger intercept identifies directional pleiotropy and biases from invalid IVs. Radial MR spots outliers, allowing for reanalysis without them. MR-PRESSO assesses for heterogeneous SNPs, while Cochran Q test, with a significance threshold of *p* < 0.05 (46), evaluates result heterogeneity. Additionally, a leave-one-out sensitivity test determined the influence of each IV on the causal estimation. In summary, our detailed analysis of GM and blood metabolites’ potential causal links to LC followed several key criteria: (1) Primary analysis significance (IVW p < 0.05 post-FDR correction); (2) Consistent direction and magnitude across five MR methods; (3) No heterogeneity or horizontal pleiotropy in MR findings; (4) Little influence of any single SNP on MR estimates. Additionally, we calculated the statistical power of our estimates using the results_binary function, incorporating Sample Size, Significance Level, Proportion of Variance Explained, Case–Control Ratio, and Odds Ratio (47).

### 2.5 Reverse Mendelian randomization analysis

To further assess the causal relationship between gut microbiota and lung cancer, we conducted reverse MR analysis considering LC as the exposure and GM or metabolite as the outcome, using the same methods and settings as the forward analysis.

### 2.6 Evaluation of genetic correlation

In MR studies, causal interpretations can be skewed by genetic links between the studied exposure and outcome. Although we excluded SNPs with direct lung cancer associations from our IVs selection, indirect SNP associations could still influence LC’s genetic structure. To confirm that observed causal effects were not muddled by genetic overlap between exposure and lung cancer, we applied LDSC regression. The threshold for genetic correlation (rg) was set at 0.05, and Genetic correlation is considered significant if the *p*-value is less than 0.05; otherwise, it is not significant. This method helps accurately assess genetic correlations and sample overlap, essential for verifying that our findings on gut microbiota, metabolites, and lung cancer stem from genuine causal connections, free from confounding by genetic coheritability.

### 2.7 Mediation analysis

Given that dysbiosis of the GM can promote the onset and progression of cancer by producing harmful metabolites (20, 48), we adopted two-step Mendelian randomization to investigate whether blood metabolites play a mediating role between the GM and LC (49). The GWAS data of blood metabolites are available in large-scale consortia or cohorts with no sample overlap with the GWASs of exposures and outcomes. Four inclusion criteria were established for each candidate mediator. First, the mediator should be causally associated with LC. Second, the mediator should have a direct causal effect on LC independently of GM. Third, GM should be causally associated with the mediator, but not vice versa. Fourth, the association of GM with the mediator and the association of the mediator with LC should be in the same direction (50). We assessed the causal impact of GM on potential mediators (*β*1) and the effect of these mediators on LC (*β*2), with the total effect (*β*3) representing the comprehensive impact of GM on LC. The magnitude of the mediated effect is calculated as the product of indirect effects (*β*1*β2), and its proportion is derived by dividing this product by the total effect, which quantifies the relative contribution or degree of influence of the gut microbiota on lung cancer through the specific mediator. We also applied the Delta method to calculate the indirect effect’s standard error (SE) (51).

### 2.8 SNP annotation

To annotate SNPs, this study employed the advanced bioinformatics tool g: SNPense,^2^ specifically designed to map human SNP rs-codes to corresponding gene names. This tool not only provides chromosomal coordinates of SNPs but also predicts potential variant effects. Notably, g: SNPense facilitates mapping only for variants overlapping with at least one protein-coding Ensembl gene, ensuring the accuracy and relevance of the annotation. All foundational data were sourced from the Ensembl genome database and WormBase ParaSite database, providing a robust and comprehensive genomic background for our SNP annotations.

### 2.9 Metabolic pathway analysis

To explore how blood metabolites might influence LC, we conducted metabolic pathway analyses with MetaboAnalyst 6.0 (52, 53),^3^ aiming to uncover LC pathogenesis. Functional enrichment and pathway analysis modules helped identify relevant metabolite groups or pathways. The Kyoto Encyclopedia of Genes and Genomes (KEGG) and the Rapid Metabolite and Pathway Mapping (RaMP) database supported our research. We set the pathway analysis significance threshold at 0.10.

All statistical analyses were conducted using the R software, version 4.3.1. For our Mendelian randomization approach, we utilized the packages “TwoSampleMR,” “MR-PRESSO,” and “PhenoScanner” which are available in R (54). Visualization of results was performed using the packages “ggplot2” and “ComplexHeatmap” also available in R.

## 3 Result

### 3.1 Two-sample Mendelian randomization analysis between microbiota and LC

**Figure 2:**
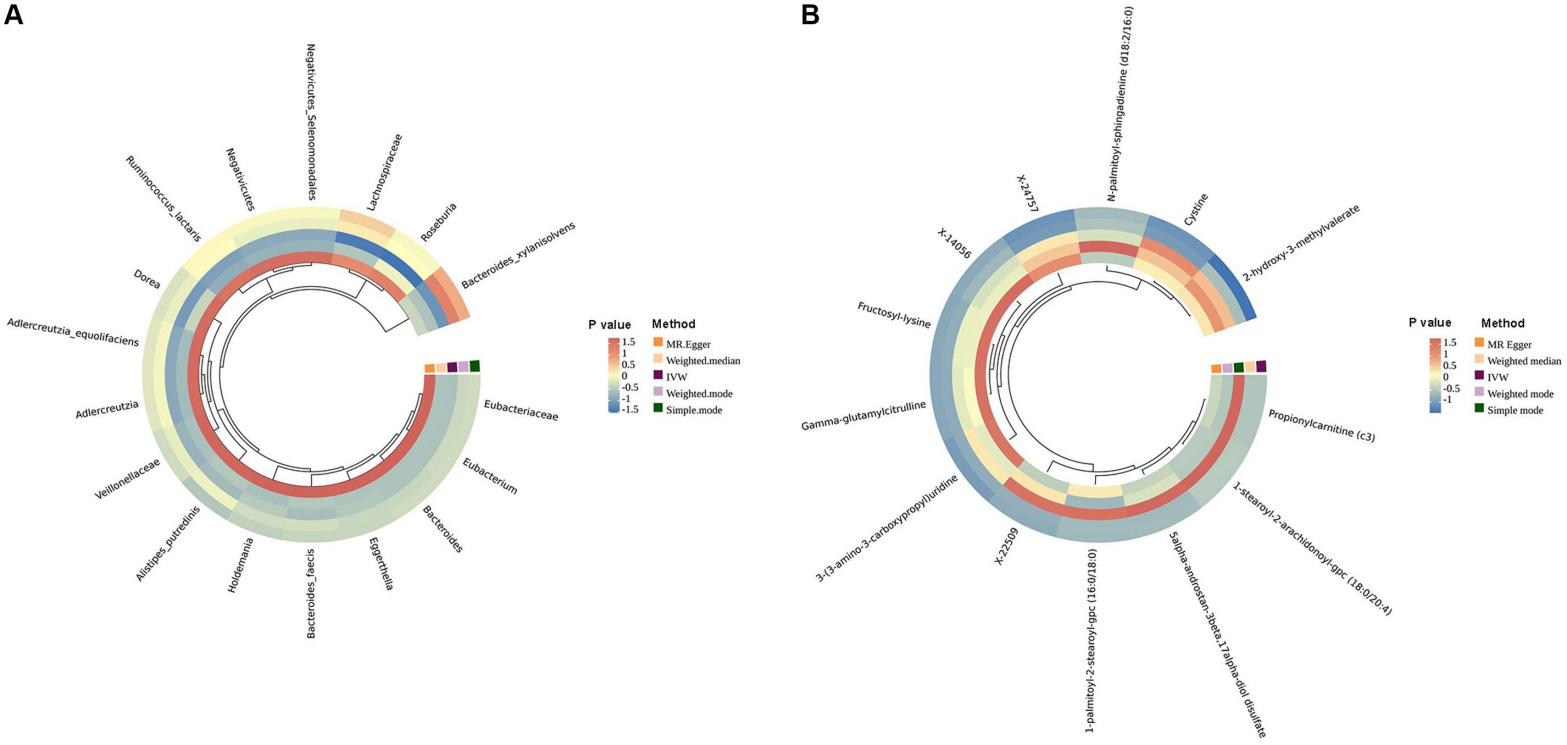
Circular heat map of lung cancer with an MR IVW *p* less than 0.05. **(A)** is the gut microbiota abundance; **(B)** is the blood metabolites.

To start with, we examined the causal relationship between genetically determined gut microbiota and the risk of lung cancer. After rigorous quality control of IVs, we identified 3,760 significant SNPs closely associated with gut microbiota. Our MR analysis then included 200 gut microbiota. The characteristics of IVs for microbial taxa and LC are detailed in Supplementary Table S1. The F statistics for all SNPs related to GM exceeded 10, demonstrating the strong power of our IVs. After excluding confounding factors (Supplementary Table S12), we investigated the associations between specific gut microbiota taxa and LC utilizing MR. Using the IVW method, we identified 17 causal relationships between GM and LC, as shown in Table 2, Figure 2A, and Supplementary Table S3. Among these 17 microbial communities, we found suggestive evidence for a causal association between genetically predicted increases in *Bacteroides_faecis* (*p* = 0.010), *Dorea* (*p* = 0.029), *Holdemania* (*p* = 0.024), *Bacteroidaceae_clarus* (*p* = 1.80E-4), *Adlercreutzia/Adlercreutzia_equolifaciens* (*p* = 0.017), *Bacteroides_xylanisolvens* (*p* = 0.003), *Negativicutes/Negativicutes_Selenomonadales* (*p* = 0.025) and *Lachnospiraceae* (*p* = 0.025) and higher risk of LC, while *Eubacteriaceae/Eubacterium* (*p* = 7.43E-06), *Alistipes_putredinis* (*p* = 0.042), *Ruminococcus_lactaris* (*p* = 0.036), *Veillonellaceae* (*p* = 0.009), *Eggerthella* (*p* = 0.022) and *Roseburia* (*p* = 0.013) were deemed negatively correlated with LC risk. After FDR correction, *Bacteroides_clarus* (OR = 1.07, 95% CI: 1.03–1.11, *p* = 0.012) remained significant. *Eubacteriaceae/Eubacterium* illustrated a protective effect on LC (OR = 0.82, 95% CI: 0.75–0.89, *p* = 0.001). Certain microbiota traits like *Enterobacteriales*, *Adlercreutzia* and *Negativicutes* shared identical IVs and were categorized similarly. *Bacteroides_clarus* persisted as positive (*p* = 0.000703) even after adjusting for relevant factors, including smoking, alcohol intake and air pollution, through MVMR analysis (Supplementary Table S4). The MR-Egger, Weighted model, Weighted median and Simple model yielded causal effect estimates with magnitudes and directions comparable to those obtained from the IVW method (see Supplementary Table S3). The variability in LC explained by these taxa, as represented by R^2^ values, ranged from 1.58 to 3.54%, while the estimated effect sizes spanned from 57.88 to 99.96%. Importantly, our findings confirmed no heterogeneity or pleiotropy, with sensitivity analyses like MR Egger reinforcing the main results’ consistency. MR PRESSO further validated the absence of pleiotropy, as detailed in Table 2, Supplementary Table S7, and Supplementary Figures S1, S2.

**Table 2:** Mendelian randomization analyses of the causal effects between gut microbiota and lung cancer.

Exposure	Method	nsnp	Odds ratio (95%CI)	P value	FDR	Q-statistics	P_h_	Egger intercept	P_intercept_
Species Bacteroides_clarus	IVW	8	1.07 (1.03–1.11)	0.0002	0.0121	7.6318	0.3662	−0.0025	0.9051
Family Eubacteriaceae	IVW	6	0.82 (0.75–0.89)	0.0000	0.0015	2.9500	0.7077	−0.0116	0.5736
Genus Eubacterium	IVW	6	0.82 (0.75–0.89)	0.0000	0.0015	2.9412	0.7091	−0.0115	0.5769
Degradation of acetylene	IVW	5	1.22 (1.10–1.35)	0.0002	0.0121	8.4969	0.0750	−0.0222	0.7943
Guanosine ribonucleotides *de novo* biosynthesis	IVW	14	0.90 (0.85–0.96)	0.0006	0.0263	21.1523	0.0700	−0.0095	0.5419
Engineered Biosynthesis of Taxadiene	IVW	10	0.93 (0.89–0.97)	0.0003	0.0136	16.8759	0.0507	0.0138	0.5368
Degradation of fucose	IVW	9	1.15 (1.07–1.24)	0.0003	0.0136	5.3251	0.7223	0.0127	0.3908

SNP, single nucleotide polymorphism; CI, confidence interval; OR, odds ratio; P_h_, *p*-value for heterogeneity; P_intercept_, *p*-value for the intercept of the MR-Egger regression.

### 3.2 Microbial pathways and lung cancer

**Figure 3:**
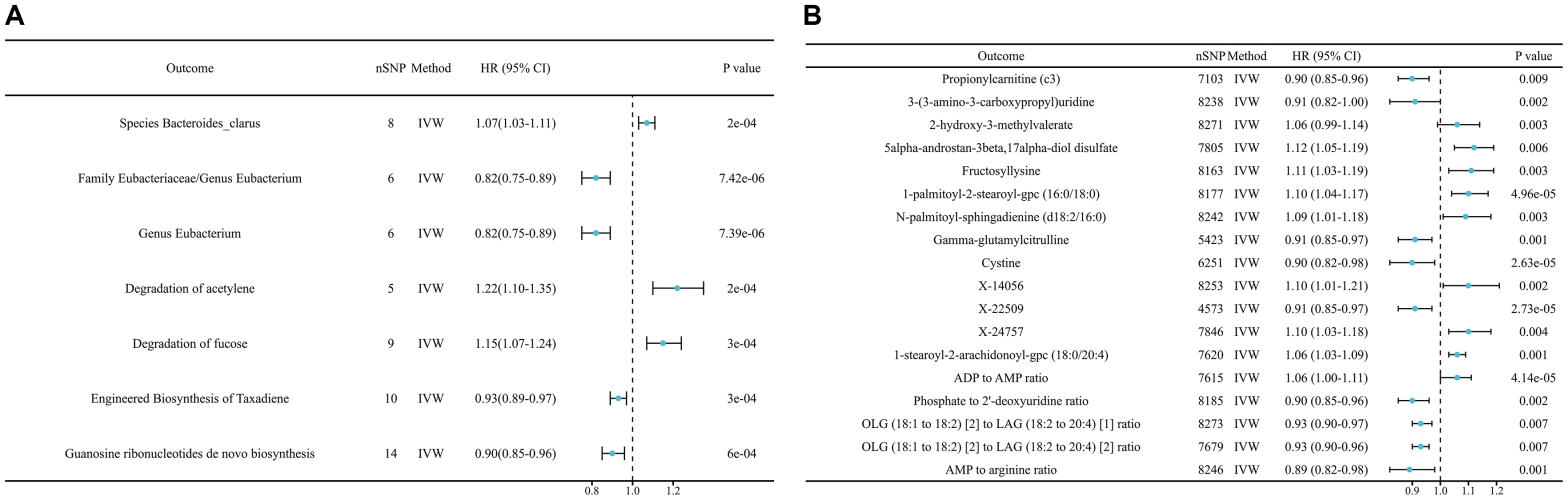
Mendelian randomization results of causal effects on lung cancer risk (*p* < 0.05). **(A)** Gut microbiome; **(B)** metabolites. ADP, Adenosine 5′-diphosphate; AMP, Adenosine 5′-monophosphate; OLG, Oleoyl-linoleoyl-glycerol; LAG, linoleoyl-arachidonoyl-glycerol.

A comprehensive set of 205 pathways was examined to determine the key targets. When solely considering the *p* value of the IVW, 30 functional pathways emerged as potential initial findings (see Supplementary Table S3). After FDR corrections, only the degradation of acetylene (OR = 1.22, 95% CI: 1.10–1.35, *p* = 0.012) and fucose (OR = 1.15, 95% CI: 1.07–1.24, *p* = 0.013) showed a positive correlation with LC incidence. In contrast, Engineered Biosynthesis of Taxadiene (OR = 0.93, 95% CI: 0.89–0.97, p = 0.013) and Synthesis of Guanosine Ribonucleotides (OR = 0.90, 95% CI: 0.85–0.96, *p* = 0.026) were negatively correlated with LC occurrence after stringent corrections (Figure 3A and Table 2). Among these, the degradation of acetylene and Synthesis of Guanosine Ribonucleotides remained significant (*p* = 0.0306, *p* = 0.0176, respectively) after MVMR validation (see Supplementary Table S4). We also performed a reverse MR analysis to explore if LC causally affects significant bacteria, following the same MR process. No significant results were found (Supplementary Table S10).

### 3.3 Mediator screening

In our study aimed at identifying potential mediators, we initially selected 1,400 metabolites to investigate their effects on LC. After performing initial filtering and completely excluding confounder-related SNPs, we identified a total of 25,879 significant SNPs. The detailed characteristics of IVs of metabolite were summarized in Supplementary Table S2. In the IVW analysis, we identified correlations between the risk of LC and 13 metabolites after rigorous FDR correction, including 10 with known chemical identities and 3 with unknown chemical identities, which were shown in Figures 2B, 3B. The 13 known metabolites were chemically assigned to the amino acid, carbohydrate, dipeptide, lipid, nucleotide and xenobiotics. They were as follows: Propionylcarnitine (OR 0.93, 95% CI: 0.90–0.97, *p* = 0.00092), 2-hydroxy-3-methylvalerate (OR 1.09, 95% CI: 1.04–1.14, *p* = 0.00034), 1-palmitoyl-2-stearoyl-gpc (16:0/18:0) (OR 1.08, 95% CI: 1.04–1.13, *p* = 4.69E-05), N-palmitoyl-sphingadienine (d18:2/16:0) (OR 1.10, 95% CI: 1.05–1.16, *p* = 0.00026), 1-stearoyl-2-arachidonoyl-gpc (18:0/20:4) (OR 1.06, 95% CI: 1.03–1.09, *p* = 0.00013), 3-(3-amino-3-carboxypropyl)uridine (OR 0.88, 95% CI: 0.83–0.94, *p* = 0.00022), 5alpha-androstan-3beta,17alpha-diol disulfate (OR 1.08, 95% CI: 1.03–1.12, *p* = 0.00064), Fructosyllysine (OR 1.09, 95% CI: 1.04–1.14, *p* = 0.00032), Gamma-glutamylcitrulline (OR 0.91, 95% CI: 0.87–0.96, *p* = 0.00010), Cystine (OR 0.88, 95% CI: 0.82–0.93, *p* = 2.63E-05), X-14056 (OR 1.12, 95% CI: 1.05–1.18, *p* = 0.000189), X-22509 (OR 0.89, 95% CI: 0.84–0.94, *p* = 2.73E-05) and X-24757 (OR 1.09, 95% CI: 1.04–1.14, *p* = 0.00036). Additionally, we observed correlations between the risk of LC incidence and the ratios of 5 pairs of metabolites after strict FDR corrrection (Figures 2B, 3B), and they are Adenosine 5′-diphosphate (ADP) to Adenosine 5′-monophosphate (AMP) ratio (OR 1.08, 95% CI: 1.04–1.12, *p* **=** 4.14E-05), AMP to arginine ratio (OR 0.88, 95% CI: 0.83–0.94, *p* = 0.00012), phosphate to 2′-deoxyuridine ratio (OR 0.93, 95% CI: 0.89–0.97, *p* = 0.00024), Oleoyl-linoleoyl-glycerol (OLG) (18:1/18:2) to linoleoyl-arachidonoyl-glycerol (LAG) (18:2/20:4) [1] ratio (OR 0.94 95% CI: 0.90–0.97, *p* = 0.00074) and OLG (18:1/18:2) to LAG (18:2/20:4) [2] ratio (OR 1.09, 95% CI: 0.92–0.98, *p* = 0.00070). In summary, IVW-derived estimates were significant (*p* < 0.05), with the direction and magnitude of IVW, MR-Egger, WM, and both weighted and simple models exhibiting consistency (see Supplementary Table S5 and Supplementary Figure S2). After the exclusion of outliers, the MR-PRESSO results further negated the presence of heterogeneous SNPs (Supplementary Table S7). The Cochran Q test (*p* > 0.05) and MR-Egger intercept test (*p* > 0.05) corroborated the absence of heterogeneity and pleiotropy (Supplementary Table S7). LOO analysis substantiated that no single SNP significantly contributed to bias in MR estimation (Supplementary Figure S4). The statistical power for all estimates exceeded 99.00% (Supplementary Table S2). The above results were obtained after excluding confounding factors (Supplementary Table S12). We also conducted a reverse MR analysis to determine if LC has a causal impact on the significant metabolites identified. Using the same MR methodology, no significant associations were found, as detailed in Supplementary Table S11. Therefore, these blood metabolites and metabolite ratios are identified as promising candidates for further investigation.

**Figure 4:**
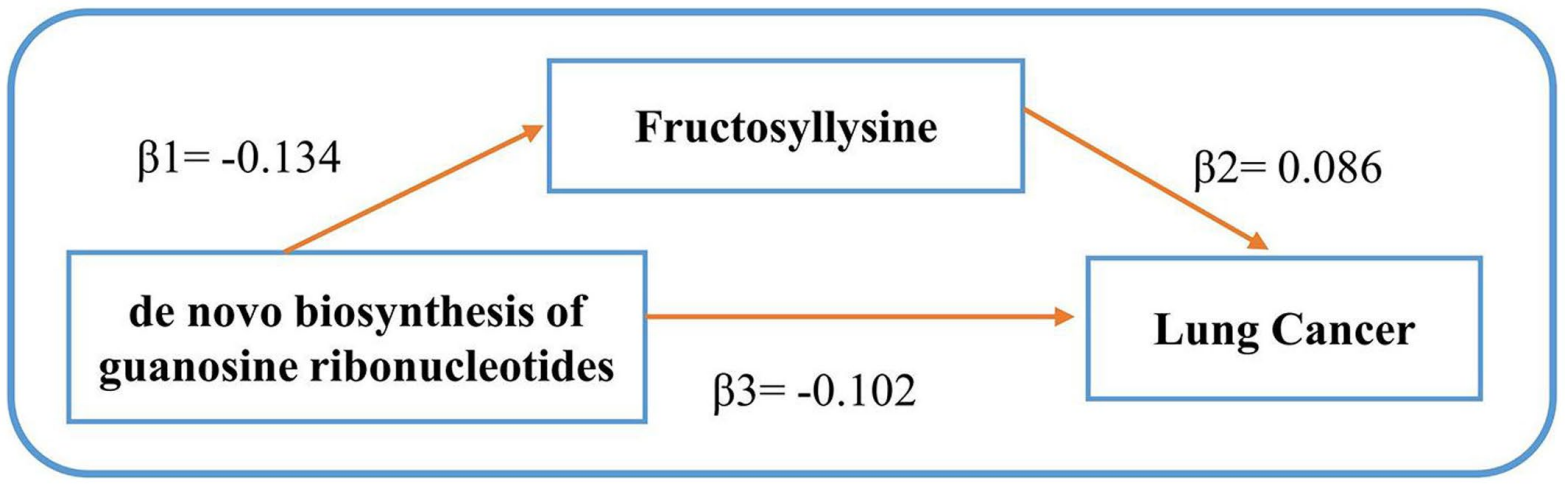
Mediating effects of gut microbiota on lung cancer. *β*1 signifies the causal influence of gut microbiota on potential mediators. *β*2 depicts the causal effect of these metabolite mediators on lung cancer. *β*3 represents the cumulative causal impact of gut microbiota on lung cancer.

Following our examination of the influence of metabolites on LC, we selected strictly screened microbial communities and microbial pathways and further explored the potential mediation effects of GM exposures on these significant mediators (refer to Figure 4). Our analysis yielded that the bacterial pathway involving the *de novo* biosynthesis of guanosine ribonucleotides was found to influence LC through its impact on Fructosyllysine, with indirect effect sizes of −0.012 (*p* = 0.0367). The detailed MR results can be seen in Supplementary Table S6. After identifying significant mediators influencing lung cancer and the subsequent effects of exposure on mediation, we assessed the mediation effect proportions. Specifically, as Figure 4 shows, the *de novo* biosynthesis of guanosine ribonucleotides demonstrated an 11.38% mediation effect on LC outcomes, mediated through Fructosyllysine. These results highlight the complex relationships between GM exposures and specific blood metabolites in influencing LC, providing a deeper understanding of the pathways involved. No significant heterogeneity or pleiotropy was observed in the analysis (see Table 3 and Supplementary Table S7).

**Table 3:** Mendelian randomization analyses of the causal effects between blood metabolites and lung cancer.

Exposure	Method	nsnp	OR (95%CI)	P value	FDR	Q-statistics	P_h_	Egger intercept	P_intercept_
Propionylcarnitine (c3)	IVW	22	0.90 (0.85–0.96)	0.0009	0.0480	23.6455	0.3105	0.0054	0.3500
3-(3-amino-3-carboxypropyl)uridine	IVW	15	0.91 (0.82–1.00)	0.0002	0.0239	19.9214	0.1326	−0.0068	0.5177
2-hydroxy-3-methylvalerate	IVW	27	1.06 (0.99–1.14)	0.0003	0.0279	29.7113	0.2797	−0.0004	0.9521
5alpha-androstan-3beta,17alpha-diol disulfate	IVW	23	1.12 (1.05–1.19)	0.0006	0.0421	27.4831	0.1934	−0.0131	0.0898
Fructosyllysine	IVW	25	1.11 (1.03–1.19)	0.0003	0.0279	36.1424	0.0532	−0.0016	0.8253
1-palmitoyl-2-stearoyl-gpc (16:0/18:0)	IVW	26	1.10 (1.04–1.17)	4.69E-05	0.0131	28.2053	0.2985	−0.0008	0.8978
N-palmitoyl-sphingadienine (d18:2/16:0)	IVW	19	1.09 (1.01–1.18)	0.0003	0.0245	15.3049	0.6409	−0.0056	0.4695
Gamma-glutamylcitrulline	IVW	24	0.91 (0.85–0.97)	0.0001	0.0204	28.9856	0.1808	0.0017	0.8899
Cystine	IVW	16	0.90 (0.82–0.98)	2.63E-05	0.0127	19.7558	0.1815	−0.0042	0.7038
X-14056	IVW	18	1.10 (1.01–1.21)	0.0002	0.0227	24.0973	0.1168	0.0071	0.3628
X-22509	IVW	18	0.91 (0.85–0.97)	2.73E-05	0.0127	27.8395	0.0468	0.0044	0.6446
X-24757	IVW	21	1.10 (1.03–1.18)	0.0004	0.0279	24.9050	0.2051	−0.0039	0.6242
1-stearoyl-2-arachidonoyl-gpc (18:0/20:4)	IVW	32	1.06 (1.03–1.09)	0.0001	0.0204	47.5948	0.0288	−0.0043	0.2430
ADP to AMP ratio	IVW	24	1.06 (1.00–1.11)	4.14E-05	0.0131	14.6195	0.9078	0.0026	0.7018
Phosphate to 2′-deoxyuridine ratio	IVW	26	0.90 (0.85–0.96)	0.0002	0.0240	36.0953	0.0702	0.0059	0.4521
OLG (18:1 to 18:2) [2] to LAG (18:2 to 20:4) [1] ratio	IVW	23	0.93 (0.90–0.97)	0.0007	0.0421	37.8373	0.0191	−0.0004	0.9487
OLG (18:1 to 18:2) [2] to LAG (18:2 to 20:4) [2] ratio	IVW	25	0.93 (0.90–0.96)	0.0007	0.0421	33.3778	0.0963	0.0010	0.8360
AMP to arginine ratio	IVW	16	0.89 (0.82–0.98)	0.0001	0.0204	15.8477	0.3922	−0.0121	0.1894

SNP, single nucleotide polymorphism; CI, confidence interval; OR, odds ratio; Ph, p-value for heterogeneity; Pintercept, p-value for the intercept of the MR-Egger regression. ADP, Adenosine 5′-diphosphate; AMP, Adenosine 5′-monophosphate; OLG, Oleoyl-linoleoyl-glycerol; LAG, linoleoyl-arachidonoyl-glycerol.

### 3.4 Evaluation of genetic correlation and directionality

LDSC analysis was conducted on the GM and metabolites identified as having significant causal relationships through MR methods. The results of the LDSC analysis unveiled the genetic correlation (rg) between various GM, metabolites, and LC (Supplementary Table S7), with *Bacteroides_clarus* showing a significant correlation with LC (Rg = −0.467, Se = 0.201, *p* = 0.020). In contrast, other gut microbiota did not exhibit significant correlations. Furthermore, LDSC-based estimates indicated a minimal genetic correlation between LC and the following metabolites: Propionylcarnitine (Rg = −0.082, Se = 0.054, *p* = 0.127), 3-(3-amino-3-carboxypropyl) uridine (Rg = 0.031, Se = 0.068, *p* = 0.652), 2-hydroxy-3-methylvalerate (Rg = 0.048, Se = 0.060, *p* = 0.419), among others. This implies that MR estimates are unaffected by shared genetic components. Additionally, the SNP-heritability of metabolites was determined based on LDSC. The SNP-heritability (proportion of variance explained by genome-wide SNPs) of these metabolites ranged from 0.0094 to 0.1681 (Supplementary Table S8). The application of the Steiger test further confirmed that the observed causal relationships between GM, genetically proxied metabolites, and LC were not influenced by reverse causation, as detailed in Supplementary Table S9.

### 3.5 SNP annotation

We annotated the SNPs at a locus-wide significance level of the three intestinal flora and identified 10 host genes that may be related to pathogenic intestinal microflora in LC patients (Table 4). In this study, the SNPs corresponding to the family *Eubacteriaceae* and the genus *Eubacterium* were identical, leading to the annotation of the same genes for both taxonomic categories.

**Table 4:** SNP annotation of intestinal flora IVs.

		id	chr	Start	End	Strand	Gene_ids	Gene_names
Genus	Eubacterium	rs1942648	7	15,736,946	15,736,946	+	ENSG00000286376	ENSG00000286376
		rs2252668	18	36,269,080	36,269,080	+	ENSG00000075643	MOCOS
		rs3898456	8	138,163,106	138,163,106	+	ENSG00000147724	FAM135B
		rs4113953		−1	−1			
		rs6021384	20	51,689,988	51,689,988	+	ENSG00000054793	ATP9A
		rs9600403		−1	−1			
Species	Bacteroides_clarus	rs1157623		−1	−1			
		rs11676681	2	33,150,881	33,150,881	+	ENSG00000049323	LTBP1
		rs1470205	3	77,267,537	77,267,537	+	ENSG00000185008	ROBO2
		rs1667938		−1	−1			
		rs450741	22	21,049,682	21,049,682	+	ENSG00000187905	LRRC74B
		rs4867972	5	170,505,906	170,505,906	+	ENSG00000182132	KCNIP1
		rs61944136	12	118,809,199	118,809,199	+	ENSG00000255814	LINC02439
		rs814760	1	110,438,037	110,438,037	+	ENSG00000224699	LAMTOR5-AS1

### 3.6 Metabolic pathway analysis

**Figure 5:**
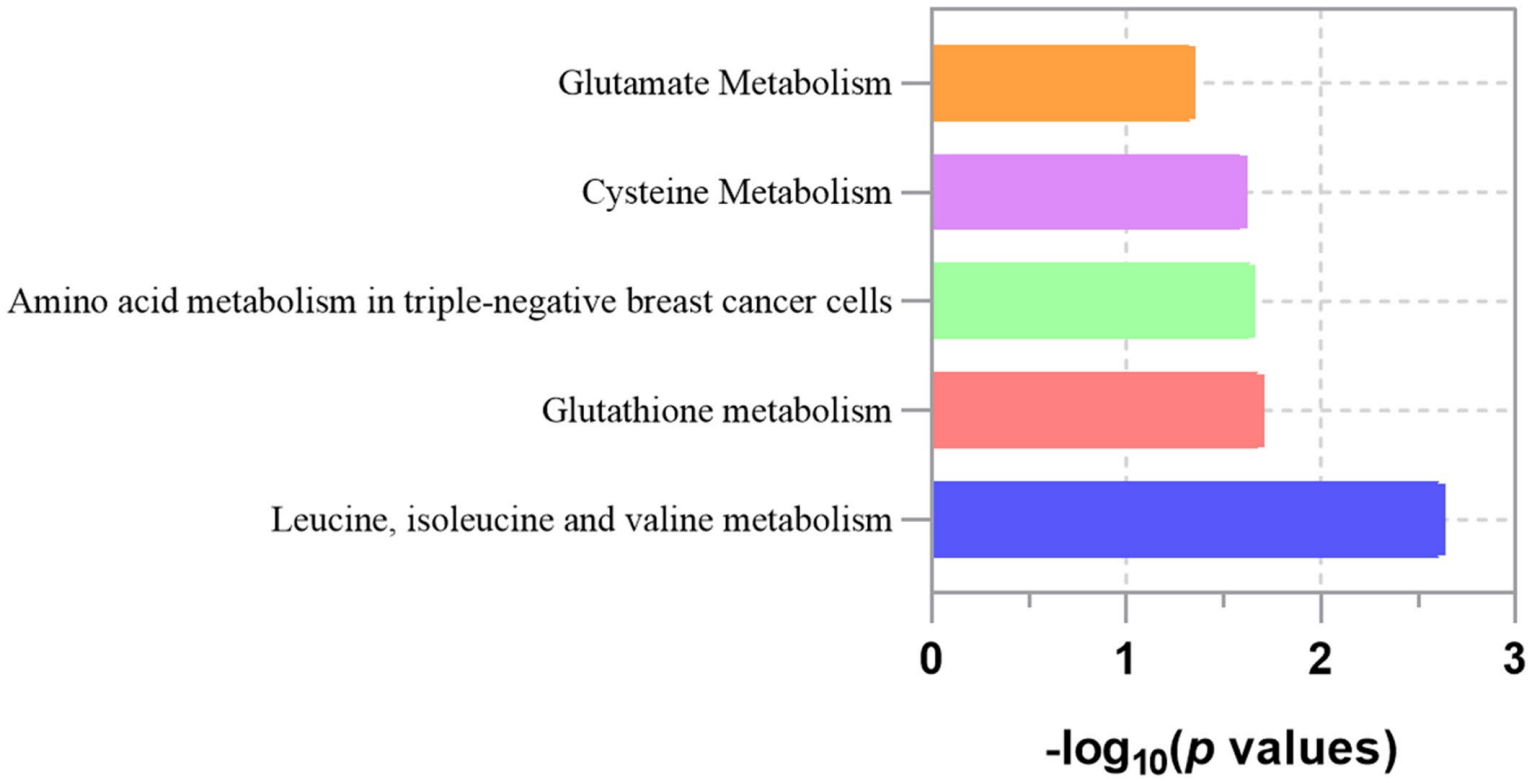
Enriched significant metabolic pathways of lung cancer.

Based on the analysis of eight known metabolites, our study identified five metabolic pathways potentially implicated in the pathogenesis of LC, as detailed in Table 5 and Figure 5. These pathways include the metabolism of leucine, isoleucine, and valine; glutathione metabolism; amino acid metabolism in triple-negative breast cancer cells; cysteine metabolism and glutamate metabolism. Importantly, glutathione metabolism and glutamate metabolism appear to be closely related to all other pathways.

**Table 5:** Significant metabolic pathways involved in lung cancer.

Metabolite pathways	Total	Expected	Hits	*p* value
Leucine, isoleucine and valine metabolism	67	0.0783	2	0.00234
Glutathione metabolism	17	0.0199	1	0.01970
Amino acid metabolism in triple-negative breast cancer cells	19	0.0222	1	0.02200
Cysteine Metabolism	21	0.0245	1	0.024300
Glutamate Metabolism	39	0.0456	1	0.044700

## 4 Discussion

Microbiome and metabolomics studies have significantly advanced our understanding of lung cancer’s pathogenic mechanisms (55, 56). The gut microbiota’s impact on lung cancer risk through metabolite interactions highlight the lung-gut axis as a critical area of research (57, 58). Through this comprehensive large-scale MR analysis, we have, for the first time, established a causal and mediating relationship between gut microbiota, metabolites, and lung cancer, addressing the gaps left by prior research constrained by confounders and reverse causation.

Our findings indicate that the s_*Bacteroides clarus* is a risk factor for LC, whereas f_*Eubacteriaceae* serves as a protective element against this disease. Significantly, *Bacteroides clarus*, a species within the g_*Bacteroides*, is a Gram-negative anaerobic bacterium that can decompose complex polysaccharides in the diet, and regulate the host immune system (59). *Bacteroides* has been proven to be a significant microbial biomarker for the non-invasive screening of colorectal neoplasms in asymptomatic individuals (60). And a notable augmentation in *Bacteroides* abundance was observed in the follow-up samples relative to the baseline samples among subjects who experienced a recurrence of adenomas (61). Moreover, *Bacteroides clarus* can coexist with other bacteria such as *Fusobacterium nucleatum* and *Peptostreptococcus anaerobius* in the intestinal epithelium, playing an important role in carcinogenesis by inducing tumor proliferation (62), enhancing inflammation (63), triggering DNA damage (64) and provide protection to tumor from immune attack (65). Indeed, the equilibrium between *Bacteroidetes* and *Firmicutes* seems to be important in the prevalence of CRC (66). The balance and composition of *Eubacteriaceae* in the gut microbiome are considered indicators of the host’s health status, with deviations linked to a range of conditions such as inflammatory bowel disease, obesity, and cancer. A research focusing on cholangiocarcinoma in mice involved high-throughput sequencing of prokaryotic 16S ribosomal DNA (67) revealed a decrease in the abundance of *Eubacteriaceae*. In contrast, the observed increase in *Eubacteriaceae* abundance in post-liver transplantation patients (68) underscores the microbiome’s recovery and the positive impact of the transplant on the patient’s gut flora. These contrasting situations emphasize the presence and balance of *Eubacteriaceae* are crucial for health. It is important to note that despite the slightly different results after MVMR adjustment, which may be due to reduced statistical power or weakened instrumental variable effects (69), the potential protective role of *Eubacteriaceae* cannot be denied.

Beyond the GM, our findings demonstrate the complex interaction between bacterial pathways and LC risk. Recent research indicates that two acetylene alcohols inhibited phosphorylation of IGF-1R*β* as well as reduced its target signaling molecules IRS-1 and PDK1, allowing inhibition of pro-survival signaling and showed anti-tumor effects (70), suggesting the gut bacterial acetylene degradation pathway may increase LC risks. The abundance of gut bacterial pathway for acetylene degradation is closely related to *Proteobacteria* and *Bacteroidetes*. It has been reported that α-, *β*-, γ-, and δ- subclasses of *Proteobacteria* all have the ability to degrade acetylene (71). Multiple clinical studies have indicated an increase in pathogens within *Proteobacteria* in fecal samples from lung cancer patients (72, 73). Understanding these dynamics is key to developing strategies that support microbiome health, thereby aiding in tumor treatment.

In the current work, we finally determined that genetically determined high levels of Cystine, Propionylcarnitine, Gamma-glutamylcitrulline and so on are associated with lower LC risk, while genetically predisposition to high levels of N-palmitoyl-sphingadienine (d18:2/16:0), 1-stearoyl-2-arachidonoyl-gpc (18:0/20:4), Fructosyllysine and so on increased risk of LC. Among the metabolites that reduce the risk of LC, cystine stands out for its antioxidative role and potential in modulating cancer progression (74). Studies show (75) that cystine supplementation rebalances redox states, enhances glutathione production, and counters drug-resistant lung cancer cell growth by reversing P-glycoprotein upregulation. These findings, combined with our research, underlining the significance of targeting the uptake and metabolic pathways of cystine in cancer therapy. In the study of propionylcarnitine, the prediction model constructed by Zhang et al. (76) proved that reduced propionylcarnitine content is significantly related to the risk of LC, which is consistent with our study results. Gamma-glutamylcitrulline, a plasma metabolite significantly correlated with glucose and lipid fluxes (77), has been previously associated with LC, along with citrulline. A study on Polish NSCLC patients found (78) that decreased level of citrulline is a clear marker of cancer. Further research (79) indicates that monitoring citrulline levels can help evaluate gut health and predict the efficacy of immune checkpoint inhibitors for lung cancer patients. Among the metabolites that contribute to the increased risk of LC, we focus on the key metabolites identified by the highest OR and the lowest *p*-values. Despite limited research on N-palmitoyl-sphingadienine (palmitoyl ceramide), evidence shows it promotes cell apoptosis, linking it to endothelial damage and LC risk (80). Ceramide was found to trigger apoptosis in lung adenocarcinoma cells by affecting the Txnip/Trx1 complex (81), which appears to contradict our results, suggesting a complex role for ceramide species. 1-palmitoyl-2-stearoyl-gpc (16:0/18:0) and 1-stearoyl-2-arachidonoyl-gpc (18:0/20:4), all belong to Phosphatidylcholine (PC). Studies have found that (82) PC isomers were associated with a shorter recurrence-free period and a greater likelihood of progressed T-factor and pleural invasion in post-surgery lung adenocarcinoma (ADC) patients who are smokers. Our study further refines the subclasses of PC, this detailed classification facilitates deeper investigation into its role in cancer progression.

The connection between microbial traits and plasma metabolic traits underscores the significant role of microbe-metabolite interactions in the carcinogenesis process (83). Building on this, our MR analysis also provided genetic evidence for the association between GM and blood metabolites. For instance, fructosyllysine (FL) is not only associated with an increased risk of LC but was also confirmed in our study to act as a mediator in the pathway of *de novo* biosynthesis of guanosine ribonucleotides, through which the gut microbiota influences LC risk. This pathway, crucial for guanosine synthesis (84)—a key molecule in RNA synthesis and LC development—impacts cancer through the ABCG transporter, reducing the proportion of side population (SP) cells in cancer (85). Additionally, activation of guanosine leads to cell cycle arrest, apoptosis, and inhibits metastasis in LC mice models (86). Understanding this mechanism is vital for creating targeted treatments, particularly for patients with KRAS mutations (87). Fructosyllysine, a key Amadori rearrangement product (ARPs) from lysine and glucose, is significantly metabolized by the gut microbiota, a factor linked to its high detection in infant feces (88). Evidence shows ARPs, including FL, trigger oxidative stress and inflammation, promoting cancer progression (89). Research by Raupbach et al. (90) on FL’s effects on colon cancer cells highlights its structure-dependent pro-inflammatory impact, underscoring the need to further study the gut microbiota’s role in FL metabolism.

A growing area of investigation in microbiome studies relevant to cancer focuses on whether dietary components mediate their beneficial health effects via the regulation of gut microbiota and metabolite. *Bacteroides*, originally studied for its role in causing human inflammatory diarrhoea, has emerged as a CRC-promoting bacterium (91). Secretion of active *B. fragilis* toxin can be suppressed by fermentable carbohydrates that are derived from plant-based diets and are constituents of healthy colonic mucus (92). In the diet of rural Africans with African Americans who consume different diets, a lower occurrence of *Bacteroides* rural Africans as compared to African Americans has been noticed. As rural Americans consume more diets based on low fiber and high fat and animal protein (93). In addition, supplementation of Wild melon (*Cucumis melo* *var. agrestis*) seed oil in the diet remarkably increased the production of fecal SCFAs and favorably altered the relative abundances of *Eubacteriaceae* at a family level (94).

Besides, environmental factors and lifestyle are also influential factors that cannot be ignored, such as air pollution. The gut is exposed to particulate matter (PM) as most of the inhaled particles are removed from the lungs to the gastrointestinal (GI) tract via mucociliary clearance. This represents a concrete manifestation of the lung-gut axis. Air pollution, particularly benzo [a] pyrene (BaP), is highly carcinogenic (95). A study explored the impact of BaP oral exposure on the gut microbiome in C57BL/6 mice (96). In the feces, compared with the control group, the relative abundance of Bacteroides was increased. Air pollution also induces changes in lipid metabolism and the redox lipidome, both of which may influence tumor development and progression (97).

Influencing GM and metabolite levels through dietary and lifestyle adjustments could be an effective prevention strategy. We suggest that lung cancer patients increase their intake of foods rich in dietary fiber, such as fruits, vegetables, and whole grains, to promote the growth of beneficial bacteria like the *Eubacteriaceae* family. These foods help produce protective metabolites such as SCFAs, which can reduce lung cancer risk (98, 99). At the same time, reducing high-sugar diets, such as candies, chocolates, smoked foods, and instant foods, and using cooking methods like moist heat can reduce the accumulation of harmful metabolites like fructosyllysine (100). Avoiding smoking may prevent the increase of harmful bacteria like *Bacteroides clarus* (101, 102), thereby reducing lung cancer risk. For individuals at high risk of lung cancer, personalized dietary and lifestyle interventions can be developed through genomic and metabolomic analyses (103) to modulate gut microbiota and reduce LC risk.

Considering the influence of the aforementioned factors, we conducted additional analyses to ensure the robustness of our results and further explored mechanisms related to host genetics. Our LDSC analysis found only *Bacteroides clarus* genetically correlated with LC, with no significant correlation for other microbes or metabolites. This is primarily because LDSC’s focus on genetic background of two triats (104) and the low heritability of gut microbiomes and metabolites (h2 *p* > 0.05), which are heavily influenced by diet, lifestyle, and medication. However, this does not dismiss a potential connection between GM, metabolites, and LC, but rather emphasizes the role of genetic and environmental factors in shaping the gut microbiome and plasma metabolites (105, 106). Future microbiome-wide association studies and metabolite-focused research may elucidate the impact of the environment on the host, thereby yielding valuable insights.

We also conducted an initial exploration of the key genes and pathways involved in the causal link between GM, blood metabolites, and LC. Our study identified 8 metabolic pathways linked to LC development, with glutathione and glutamate metabolism playing key roles (107, 108). Abnormal glutathione metabolism expression affects oxidative stress, apoptosis, and ferroptosis in NSCLC cells (109, 110). Recent studies have pointed out that the glutathione metabolism core SMS gene was highly enriched in M2 Macrophages in lung adenocarcinoma (111). Furthermore, glutamine metabolism is involved in biosynthesis and redox reactions and has been proven to be the key metabolic pathway contributing to the chemo-immunotherapy response in advanced NSCLC patients (112). Single-cell RNA-seq data analysis revealed that the glutamine metabolism gene scores of tumor cells were significantly higher than those of CD8T cells, and glutamine metabolism inhibitor could promote the proliferation of CD8T cells (113).

Studies have shown that GM and host gene expression vary during the disease process, with certain microbes stimulating oncogene transcription in NSCLC cells (114), highlighting the importance of GM-host gene interactions in lung cancer development. We linked 10 host genes, including ROBO2, to gut microbe abundance in LC, with studies (115) showing a correlation between ROBO2 and oral microbiota levels. During tumor formation, advanced sequencing techniques suggest ROBO2’s immunosuppressive function may influence the tumor microenvironment, contributing to the progression of various cancers (116–118). Interestingly, further research indicates that ROBO family-related signaling pathways may be involved in macrophage immune responses by inducing cytoskeletal changes in macrophages (119). These findings suggest that immune cells and the TME are critical factors in the disease process (120), and advanced methods like the scPagwas method (121), combined with immune cell scRNA-seq data, could be used for further exploration.

Our research stands out for its comprehensive approach. First, we analyzed 412 gut microbiotas and 1,400 blood metabolites, alongside integrating data from over 170,000 individuals from two lung cancer databases for enhanced statistical solidity. Second, to overcome limitations like reverse causality, we conducted strict MR analyses, employing SNPs as IVs, with consistency checks across five MR estimates and sensitivity analyses. Besides the host’s genetic characteristics, we considered the impact of confounding factors and applied MVMR to ensure the robustness of the results. Moreover, we employed Two-sample MR and mediation analysis to explore linear and possible nonlinear relationships. Third, apart from MR analysis, we assessed the heritability of IVs through LDSC, explored metabolite pathways related to lung cancer risk, and conducted a preliminary exploration of the gut microbiome genome.

Nonetheless, the current MR study has limitations, including the exclusive inclusion of European participants. Differences in diet, lifestyle, environmental exposures, and genetic backgrounds can influence the genetic architecture and prevalence of specific GM and metabolites across ethnicities. These differences could result in distinct causal pathways and interactions between GM, metabolites, and LC risk in non-European populations. Additionally, although we identified gene-gut microbiome associations via SNP annotation, the diagnostic and prognostic relevance of these lung cancer-specific links requires further clinical validation due to the scarcity of existing studies. Furthermore, we were unable to include detailed dietary data. The lack of comprehensive dietary information prevents us from accounting for the potential effects of various dietary patterns on gut microbiota composition and metabolism. Lastly, the inability to dynamically capture the changes in GM, metabolites, and LC development limits our understanding of how these relationships change over time and prevents us from accurately identifying the specific time points at which they interact during disease progression.

Future studies should include a more diverse range of participants to capture the full spectrum of genetic and environmental factors affecting these relationships. Additionally, if sufficient sample sizes are available, we plan to explore different lung cancer subtypes, such as small cell lung cancer, lung adenocarcinoma, and others, to offer more precise prevention and treatment strategies. We also suggest considering the collection of detailed dietary data to better understand the impact of dietary patterns on gut microbiota and their metabolites. And we plan to collect longitudinal data, including regular fecal and blood samples from participants, to monitor the dynamic changes in gut microbiota, metabolite levels, and lung cancer development. Furthermore, we will consider incorporating more environmental and lifestyle factors, such as medicine usage and exercise, through questionnaires and community collaborations. This comprehensive approach aims to elucidate how the relationships between gut microbiota, blood metabolites, and lung cancer evolve over time and in response to external factors.

## 5 Conclusion

Our MR and genetic analysis revealed causal links between three microbial communities, four GM pathways, thirteen metabolites, and LC, also investigating how metabolites mediate the relationship between the GM and LC. This study highlights the importance of considering gut microbiota and their metabolites in the clinical prevention and treatment of lung cancer, and proposes that interventions related to gut microbiota and metabolites could include promoting healthy lifestyles such as avoiding smoking, regulating diet by increasing the intake of foods rich in dietary fiber and reducing high-sugar diets, and taking precautions in environments with poor air quality, such as wearing masks.

## Data Availability

The datasets presented in this study can be found in online repositories. The names of the repository/repositories and accession number(s) can be found in the article/Supplementary material.

## Footnotes

^1^https://www.ebi.ac.uk/gwas/

^2^https://biit.cs.ut.ee/gprofiler/snpense

^3^https://www.metaboanalyst.ca/
