# Supplementary figures and images for: Gut microbiota influence on lung cancer risk through blood metabolite mediation: from a comprehensive Mendelian randomization analysis and genetic analysis
Source: Front Nutr. 2024 Sep 11;11:1425802. doi: 10.3389/fnut.2024.1425802 (PMC11423778; doi:10.3389/fnut.2024.1425802)

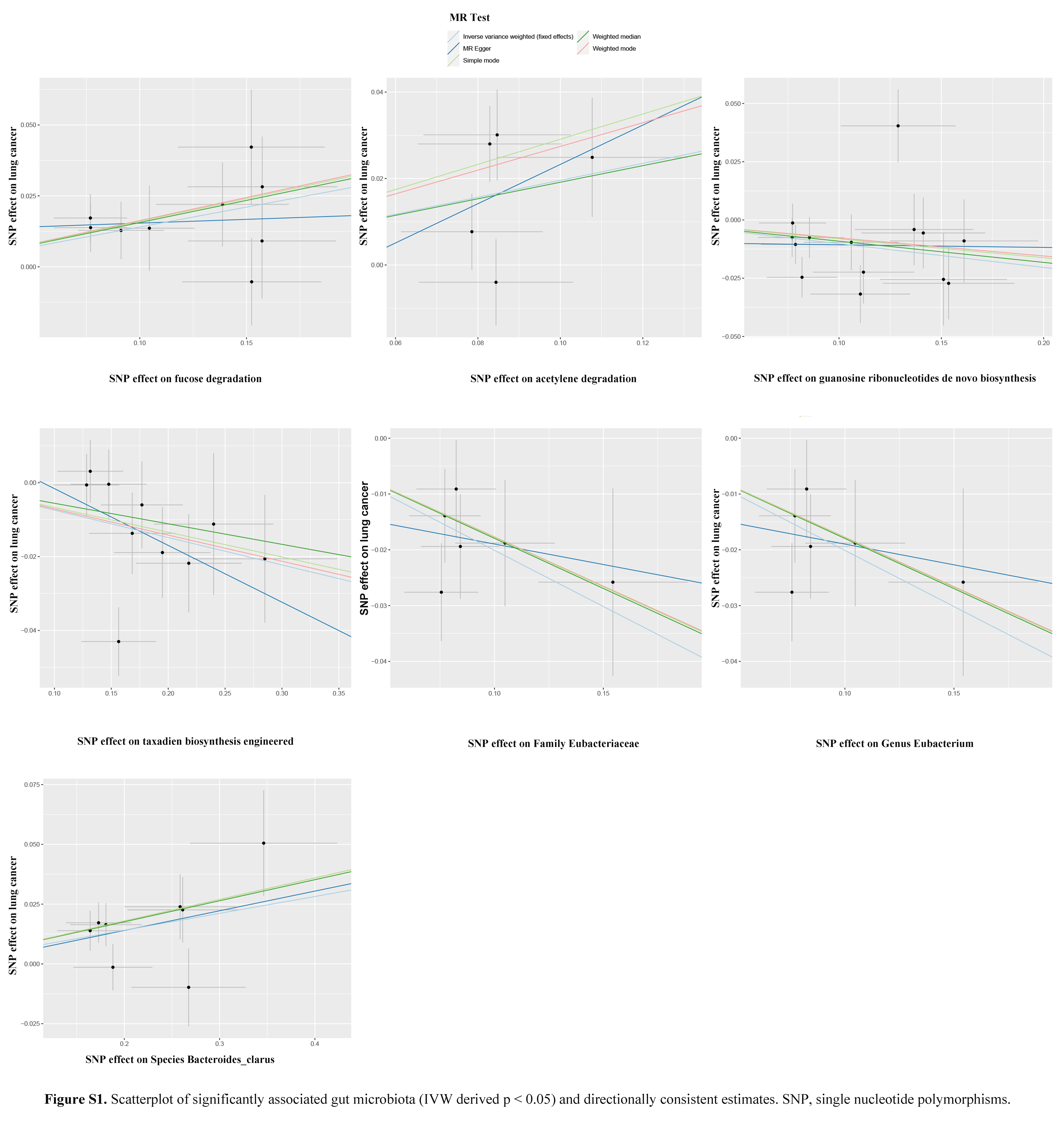

Supplement: Supplementary file 1 [file Image_1.JPEG]

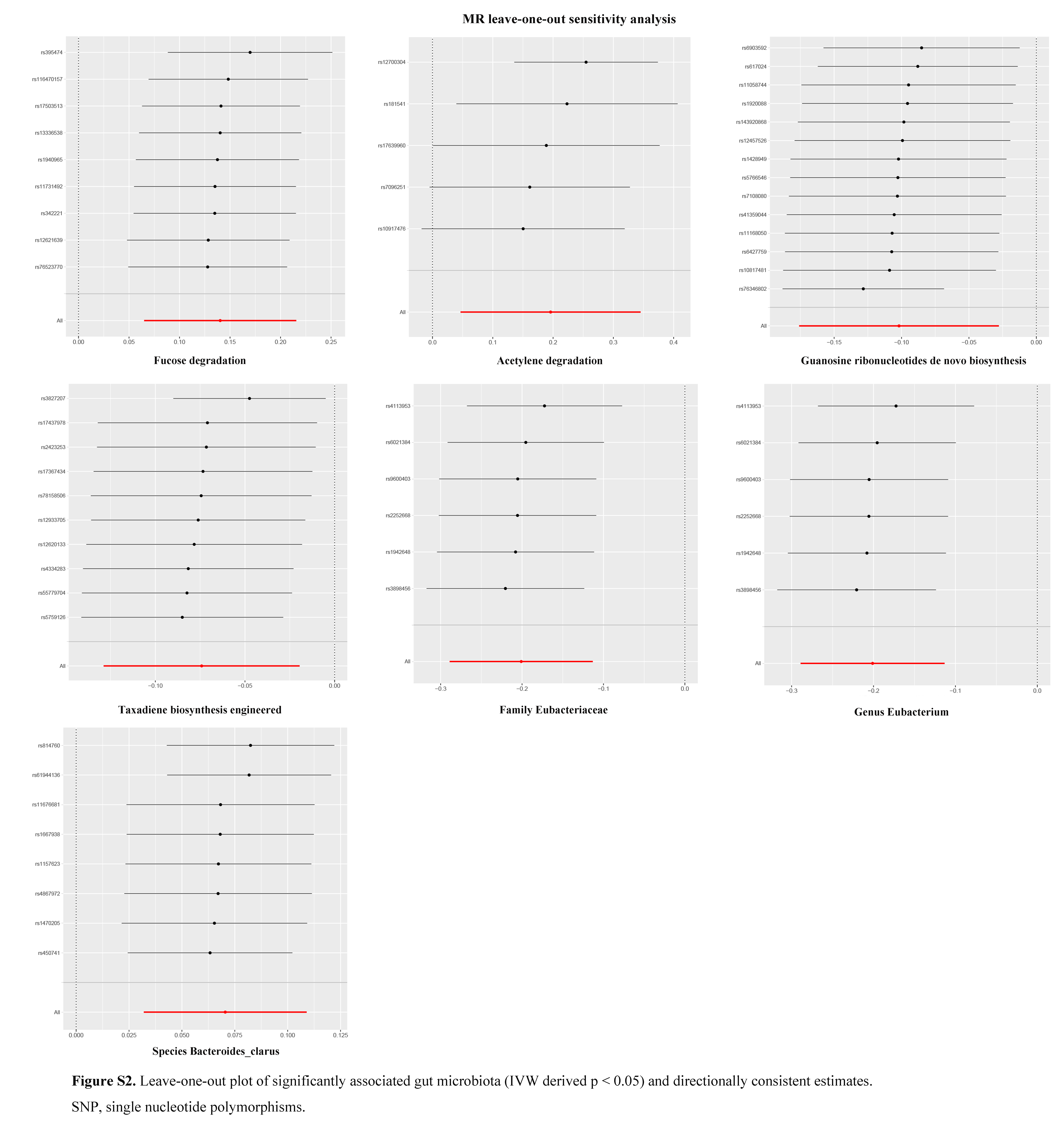

Supplement: Supplementary file 2 [file Image_2.JPEG]
